# Missing the guidewire: an avoidable complication

**DOI:** 10.1186/1755-7682-3-21

**Published:** 2010-09-25

**Authors:** Hesham R Omar, Ahmed Fathy, Devanand Mangar, Enrico Camporesi

**Affiliations:** 1 Department of Cardiovascular Medicine, Cairo University Hospital, Cairo, Egypt; 2 Tampa General Hospital, Tampa, Florida, USA; 3 Department of Cardiovascular Medicine, National Heart Institute, Cairo, Egypt; 4 Department of Anesthiology, Tampa General Hospital, Tampa, Florida, USA; 5 Department of Surgery/Anesthesiology, Department of Molecular Pharmacology and Physiology, University of South Florida, Tampa, Florida, USA

## Abstract

Central venous catheterization is an imperative tool in the critically ill patient to administer fluids, medications and for monitoring the central venous pressure. This procedure is associated with a variety of complications, some of which can be life threatening. In this brief report, we are addressing one of the rare complications of central venous catheterization which is missing the guidewire. We also described several precautions to avoid this complication as well as modifications in the guidewire to prevent its escape.

## Case Report

**Figure 1:**
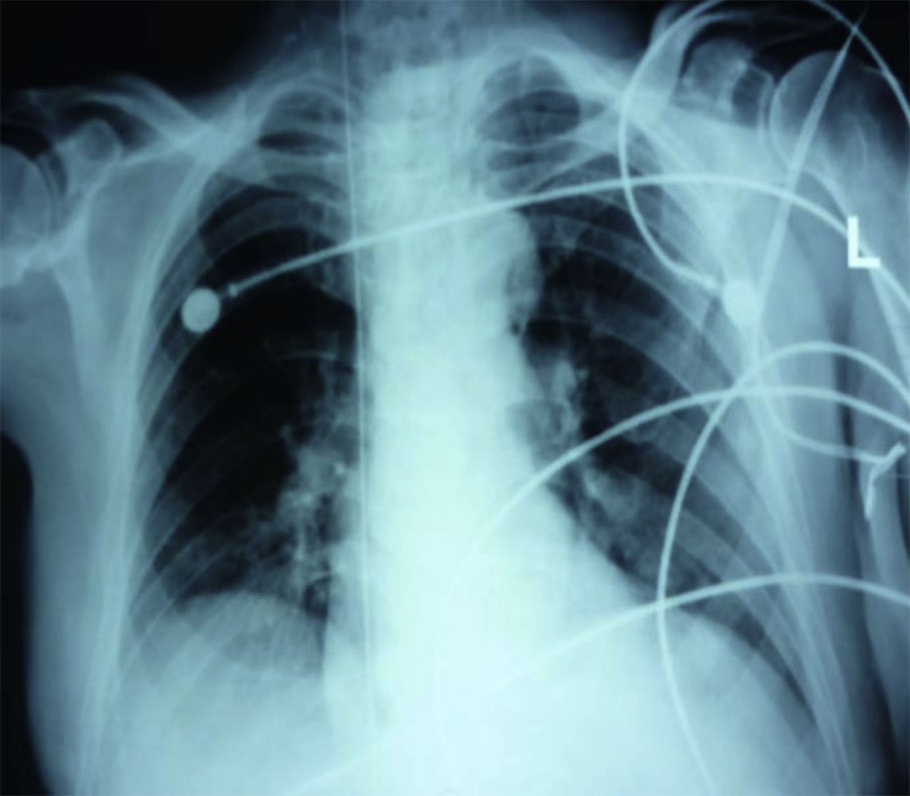
**Antero-posterior Chest X-ray revealing the guidewire passing through the internal jugular vein, superior vena cava, right atrium and inferior vena cava**.

An 80 year old male presented to the ER after collapsing at a nursing home. Cardiopulmonary resuscitation was performed and he was admitted to the ICU. The patient was shocked, so a central line was inserted for both infusion of vasopressors and monitoring of the central venous pressure. A posterior approach was used for cannulation of the internal jugular vein. The guidewire was introduced smoothly after positive aspiration of venous blood. The dilator was advanced over the guide-wire to create a track for the catheter. On removing the dilator, the guidewire was missing. Chest X-ray was immediately performed and the wire was found as shown in figure 1. The guide-wire was then removed surgically by the vascular surgery team.

Central venous catheterization is an imperative tool for many critically ill patients whether to administer fluids, drugs or measure the venous pressures. The complication rate of this procedure maybe as high as 12% [1] In this report we are addressing one of the rare complications following central venous catheterization which is missing the guidewire. Although this complication has been rarely reported, we assume that this is underestimated due to medicolegal reasons. Usually the loss of a complete guide-wire passes uncomplicated; however complications might arise due to embolization from fragmentation of the guidewire or from a thrombus formed over the guidewire. Other possible complications include vascular injury and cardiac arrhythmias. Interventional radiology is the method of choice for wire removal using either a gooseneck [2] snare or a Dormier basket [3]

## 'Physician tips to avoid this complication

1. A quiet environment is required to minimize distraction to the operator.

2. Ensure adequate length of the guidewire outside the patient sufficiently exceeding the length of the catheter and the dilator.

3. The tip of the guidewire should be kept in hand of the operator or assistant at all times throughout the procedure.

4. Make sure that the guidewire is visible at its proximal end before advancing the catheter or dilator.

5. After the guidewire is inserted, and the dilation is done, clamp a hemostat to the proximal end, to secure it stays outside the body.

6. Ensure the presence of the guidewire in the set after completing the procedure.

7. Post insertion X-ray should be immediately performed and read by the line inserter, as often a second reader might not notice, and/or the inserted catheter might obscure the vision of the wire.

## We suggest the following techniques to avoid this complication

1. Placing a landmark over the wire that serves as a warning sign to avoid advancing the wire beyond this level. The distance between the skin puncture site and the cavo-atrial junction was calculated by Andrew et al [4] as shown in table 1.

**Table 1:** Demonstrating the distance between the skin puncture site and cavo-atrial junction in various central venous catheterization approaches.

Right Internal Jugular Vein to Atrio-caval Junction	16.0 cms
Right Subclavian Vein to Atrio-caval Junction	18.4 cms

Left Internal Jugular Vein to Atrio-caval Junction	19.1 cms

Left Subclavian Vein to Atrio-caval Junction	21.2 cms

**Figure 2:**
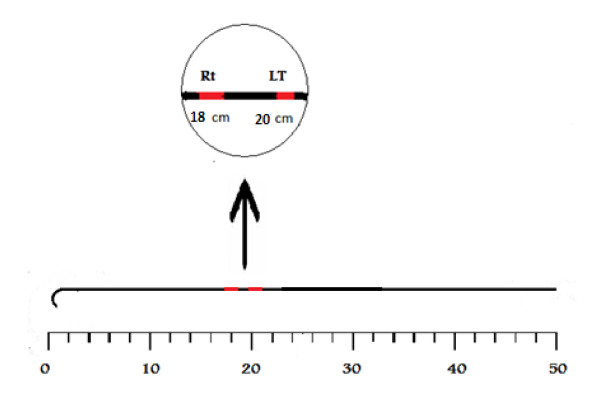
**Revealing the presumed guide-wire with 2 landmarks, the first is 18 cms away from the curved end for right sided approaches and the second is 20 cms away from the curved end for left sided approaches**. The wire should not be advanced beyond these landmarks.

We think that it is reasonable to advance the guide-wire no more than 18 cms for right sided approaches and 20 cms for left sided approaches. This will also prevent ventricular arrhythmias that can occur from introduction of excessive lengths of the guidewire. We assume that the longer the length of the wire outside the patient, the less the incidence of its loss. The guidewire should have markings corresponding to these 2 points beyond which the guide-wire should not be advanced as shown in figure 2.

**Figure 3:**
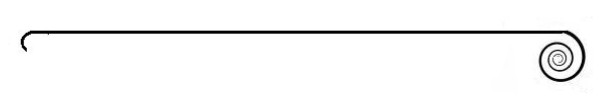
**Revealing the presumed design of the wire with its distal end coiled instead of being straight to prevent slippage of the wire**.

2. We suggest another design for the guidewire with its distal end coiled up several times instead of being straight to prevent accidental slipping of the wire through the needle or the dilator as shown in figure 3. However, this coiled end should be kept malleable enough to allow sliding of the needle, catheter and dilator over it.

## Consent

Written informed consent was obtained from the patient's next of kin for publication of this case report. A copy of the written consent is available for review by the Editor-in-Chief of this journal

## Competing interests

The authors declare that they have no competing interests.

## Authors' contributions

HO and AF were responsible for drafting the manuscript. DM and EC have made critical revisions to the manuscript. All authors have read and approved the final manuscript.
